# [Retracted] MicroRNA‑338‑3p suppresses cell proliferation, migration and invasion in human malignant melanoma by targeting MACC1

**DOI:** 10.3892/etm.2024.12533

**Published:** 2024-04-08

**Authors:** Chunhua Zhang, Hui Li, Junling Wang, Jibei Zhang, Xiaoqian Hou

Exp Ther Med 18:997–1004, 2019; DOI: 10.3892/etm.2019.7644

Following the publication of this paper, it was drawn to the Editor’s attention by a concerned reader that certain of the western blot data in Fig. 4C and the Transwell cell invasion assay data shown in Fig. 5C on p. 1002 were strikingly similar to data appearing in different form in other articles written by different authors at different research institutes that had already been published elsewhere prior to the submission of this paper to *Experimental and Therapeutic Medicine* (several of which have already been retracted). Moreover, identical data which were intended to show the results from differently performed experiments were identified comparing the scratch-wound assay data in Figs. 3A and 5B.

In view of the fact that the abovementioned data had already apparently been published previously, the Editor of *Experimental and Therapeutic Medicine* has decided that this paper should be retracted from the Journal. The authors were asked for an explanation to account for these concerns, but the Editorial Office did not receive a reply. The Editor apologizes to the readership for any inconvenience caused.

